# Microbial Community Dynamics and Activity Link to Indigo Production from Indole in Bioaugmented Activated Sludge Systems

**DOI:** 10.1371/journal.pone.0138455

**Published:** 2015-09-15

**Authors:** Yuanyuan Qu, Xuwang Zhang, Qiao Ma, Jie Deng, Ye Deng, Joy D. Van Nostrand, Liyou Wu, Zhili He, Yujia Qin, Jiti Zhou, Jizhong Zhou

**Affiliations:** 1 State Key Laboratory of Fine Chemicals, Key Laboratory of Industrial Ecology and Environmental Engineering (Ministry of Education), School of Environmental Science and Technology, Dalian University of Technology, Dalian, 116024, China; 2 Institute for Environmental Genomics (IEG), Department of Microbiology and Plant Biology, University of Oklahoma, Norman, Oklahoma, 73019, United States of America; 3 Earth Sciences Division, Lawrence Berkeley National Laboratory, Berkeley, California, 94720, United States of America; 4 State Key Joint Laboratory of Environment Simulation and Pollution Control, School of Environment, Tsinghua University, Beijing, 100084, China; Oak Ridge National Lab, UNITED STATES

## Abstract

Biosynthesis of the popular dyestuff indigo from indole has been comprehensively studied using pure cultures, but less has been done to characterize the indigo production by microbial communities. In our previous studies, a wild strain *Comamonas* sp. MQ was isolated from activated sludge and the recombinant *Escherichia coli*_*nagAc*_ carrying the naphthalene dioxygenase gene (*nag*) from strain MQ was constructed, both of which were capable of producing indigo from indole. Herein, three activated sludge systems, G1 (non-augmented control), G2 (augmented with *Comamonas* sp. MQ), and G3 (augmented with recombinant *E*. *coli*_*nagAc*_), were constructed to investigate indigo production. After 132-day operation, G3 produced the highest yields of indigo (99.5 ± 3.0 mg/l), followed by G2 (27.3 ± 1.3 mg/l) and G1 (19.2 ± 1.2 mg/l). The microbial community dynamics and activities associated with indigo production were analyzed by Illumina Miseq sequencing of 16S rRNA gene amplicons. The inoculated strain MQ survived for at least 30 days, whereas *E*. *coli*_*nagAc*_ was undetectable shortly after inoculation. Quantitative real-time PCR analysis suggested the abundance of naphthalene dioxygenase gene (*nagAc*) from both inoculated strains was strongly correlated with indigo yields in early stages (0–30 days) (*P* < 0.001) but not in later stages (30–132 days) (*P* > 0.10) of operation. Based on detrended correspondence analysis (DCA) and dissimilarity test results, the communities underwent a noticeable shift during the operation. Among the four major genera (> 1% on average), the commonly reported indigo-producing populations *Comamonas* and *Pseudomonas* showed no positive relationship with indigo yields (*P* > 0.05) based on Pearson correlation test, while *Alcaligenes* and *Aquamicrobium*, rarely reported for indigo production, were positively correlated with indigo yields (*P* < 0.05). This study should provide new insights into our understanding of indigo bio-production by microbial communities.

## Introduction

Indigo is one of the most popular dyestuffs widely used in dyeing by the textile, food and pharmaceutical industries. Although it used to be produced by extraction of plants such as *Indigofera* and *Polygonum tinctorium* [1,2], indigo is mainly produced by chemical synthesis. Because the toxic wastewater generated by chemical processes contains aniline, cyanide and high levels of chemical and biological oxygen demands (COD and BOD), researchers have been trying to develop greener methods for indigo production [3,4]. Microbial production of indigo could be a competitive alternative owing to the environmentally-friendly nature [3].

Since 1983, a lot of indigo-producing bacterial strains, belonging to the genera *Pseudomonas* [1,5–8], *Rhodococcus* [9], *Sphingomonas* [10], *Acinetobacter* [11,12] and *Comamonas* [13], have been isolated from soils, polluted sediments, intertidal sediments and activated sludge. The enzymes responsible for indigo bio-production (mono- or di-oxygenases) have been used for the construction of genetically engineered microorganisms (GEMs) [14–17], and the mechanisms of indigo production from indole by both wild strains and GEMs have been studied with the help of advanced chemical analytical methods [5,16,18]. In recent study we found that microbial communities stimulated by different aromatics could also produce indigo from indole, but the indigo production was dissimilar among different groups [19]. However, compared with the pure-culture studies, the role of microbial communities in indigo production still remains poorly understood.

The use of microbial communities in an industrial process has many advantages over the use of pure cultures. For example, microbial communities can perform complicated functions that a single population cannot, and they are more stable and resilient to complex environmental conditions [20–22]. In addition, bioaugmentation with specific strains may further improve the performance of indigenous microbial communities, which has been successfully applied in a variety of bioremediation processes [23,24]. However, the role and fate of the introduced inocula are still debated [25]. With the rapid development of high-throughput sequencing technologies, it is possible to obtain a detailed picture on the composition, structure and dynamics of microbial communities [26–29]. Such information may have important implications on understanding microbial communities for indigo bio-production from indole.

In previous studies we showed that the wild strain *Comamonas* sp. MQ (a genus within *Betaproteobacteria*), the recombinant *Escherichia coli*_*nagAc*_ carrying the naphthalene dioxygenase gene (*nag*) from strain MQ, and the activated sludge systems were capable of producing indigo from indole [13,17,19]. In this study, we tried to address the following questions: (i) Does inoculation of strain MQ/*E*. *coli*_*nagAc*_ affect the composition of the indigenous microbial community and alter the efficiency of indigo production? (ii) What microorganisms play key roles in indigo production in these AS systems? To answer these questions, three AS systems, i.e. non-augmented AS (G1), AS plus *Comamonas* sp. MQ (G2) and AS plus *E*. *coli*_*nagAc*_ (G3), were prepared to examine indigo biosynthesis from indole, and the microbial communities were analyzed using Illumina MiSeq sequencing technology. This study should provide a new insight to understand the microbial production of indigo.

## Materials and Methods

### Bacterial strains

*Comamonas* sp. MQ (CGMCC No. 6865) was isolated from activated sludge of a local sewage farm (Dalian, China) [13]. The activated sludge used in this study was collected from Chunliu River Wastewater Treatment Plant (Dalian, China) under the permission of Dalian Drainage Department, and the field studies did not involve endangered or protected species. The recombinant *E*. *coli*_*nagAc*_ carrying the naphthalene dioxygenase gene (GeneBank ID JN655512) from strain MQ was constructed previously [17]. Strain MQ was cultivated in Luria-Bertani (LB) medium with 300 mg/l naphthalene and incubated at 30°C with continuous shaking until the bacteria reached the late logarithmic phase of growth. The recombinant *E*. *coli*_*nagAc*_ was cultured according to the methods described in the previous study [17]. Both strains were harvested by centrifugation at 8,000 × *g* for 5 min. The cells were washed twice with sterile sodium phosphate buffer (PBS, 0.2 M, pH 7.0), and the cell pellets were used for inoculation.

### Experimental design and operation conditions

Three sequencing batch reactors (SBRs) were simulated with 250-ml flasks containing 100 ml synthetic wastewater, which consisted of 6 g/l Na_2_HPO_4_, 3 g/l KH_2_PO_4_, 0.5 g/l NaCl, 1 g/l NH_4_Cl, 0.011 g/l CaCl_2_, 0.24 g/l MgSO_4_ and 0.2 g/l naphthalene. The SBRs were seeded with the activated sludge (0.54 g, dry weight at 105°C), and domesticated with the wastewater for 15 days. Then, the SBRs were inoculated with different bacteria: (1) group 1 (G1), non-augmented SBR; (2) group 2 (G2), augmented SBR with *Comamonas* sp. MQ (0.22 g, dry weight); (3) group 3 (G3), augmented SBR with recombinant *E*. *coli*_*nagAc*_ (0.24 g, dry weight). The whole operation process was divided into three stages: T1 stage (0–30 d), indole was 73–85 mg/l; T2 stage (30–81 d), indole was 168–185 mg/l; and T3 stage (81–132 d), indole was 277–290 mg/l. Each operation cycle of SBR was performed for 72 h, including 2 h filling, 66 h reacting, 2 h settling and 2 h decanting. At the end of each SBR operation cycle, samples were taken to monitor the yields of indigo and the residual concentrations of indole. The pure culture controls were performed by inoculating the same amount of strain MQ and recombinant *E*. *coli*_*nagAc*_ into the synthetic wastewater, respectively, and the operation processes were carried out under the identical conditions as the AS systems.

### Chemical analysis

The concentrations of indole and indigo were measured using high performance liquid chromatography (HPLC) (Shimadzu LC20A; Thermo Hypersil ODS-2 column, 5 μm, 250×4.6 mm). The pigments were also analyzed by HPLC-mass spectroscopy (MS) to identify the products. HPLC and MS were conducted as described previously [13,17].

### DNA extraction, PCR amplification, and sequencing

During the operation, activated sludge samples were taken concurrently from the three reactors for high-throughput sequencing, and 11 samples were collected from each group (Table A in S1 File). The genomic DNA was extracted using the protocol previously described [30]. DNA concentration was determined by Pico Green assay using a FLUOstar OPTIMA fluorescence plate reader (BMG Labtech, Germany), and each DNA sample was diluted to 10 ng/μl for PCR amplification. The V4 region of the 16S rRNA gene was amplified using the methods previously described [19]. High-throughput sequencing of the 16S rRNA gene was conducted on Illumina MiSeq platform for 300 cycles at the Institute for Environmental Genomics, University of Oklahoma.

### Data analysis

After sequencing, the bar-codes and primers were removed, and the paired-end (PE) reads were overlapped using the Flash program to assemble the final V4 tag sequences [31]. The low-quality fragments were all eliminated, including the sequences without exactly matching the forward primer, the sequences containing ambiguous reads (N), and the variable tags shorter than 240 bp. The clean sequences were then subjected to Chimera detection by UCHIME [32]. Each sample was randomly re-sampled and normalized at 27,530 sequences. Operational taxonomic units (OTU) were classified at 97% similarity level using CD-HIT [33], and the reads from singleton OTUs were removed. In addition, the taxonomic assignment of OTUs was performed by RDP classifier with a confidence threshold of 50% [34]. The Shannon index (H), Pielou’s evenness index (J), species richness estimator of Chao1 and rarefaction curves were analyzed by Mothur for each sample [35]. The detrended correspondence analysis (DCA) was performed by Canoco 4.5 [36]. The dissimilarity tests based on Bray-Curtis similarity distance matrices were performed by the Vegan package in R 3.1.2 (http://www.r-project.org/), including permutational multivariate analysis of variance (Adonis), analysis of similarity (ANOSIM) and multiresponse permutation procedure (MRPP) [37]. A phylogenetic tree was constructed by MEGA 5.1 using neighbor-joining algorithm with 1,000 bootstrap replicates. The 10 most abundant genera in each group were depicted in a heat map conducted by R 3.1.2. Pearson correlation was calculated to determine the relationship between the relative abundances of microbial taxa and indigo yields. The sequencing data have been deposited into the NCBI Sequence Read Archive database under the accession number SRX897059.

### Quantitative real-Time PCR assays

The naphthalene dioxygenase gene (*nagAc*) of *Comamonas* sp. MQ was selected for qPCR assays, which were conducted in triplicate using TaKaRa PCR Thermal Cycler Dice Real Time System (TaKaRa, China) with the primer set nagF (5’-CAG CGC ACT TTC GGA ACC-3’) and nagR (5’-CTG GTA GGC GCG GTA AAA G-3’). The qPCR mixture (25 μl) contained 12.5 μl of SYBR Premix Ex *Taq* (TaKaRa, China), 1 μl of each primer (10 μM), and 2 μl of template DNA. The thermal profile included 30 s of initial denaturation at 95°C, and 40 cycles of 5 s at 95°C and 30 s at 60°C. Amplicons were visualized and checked by electrophoresis on agarose gel (1.5%, w/v).

## Results

### Indigo production from indole in the AS systems

**Fig 1:**
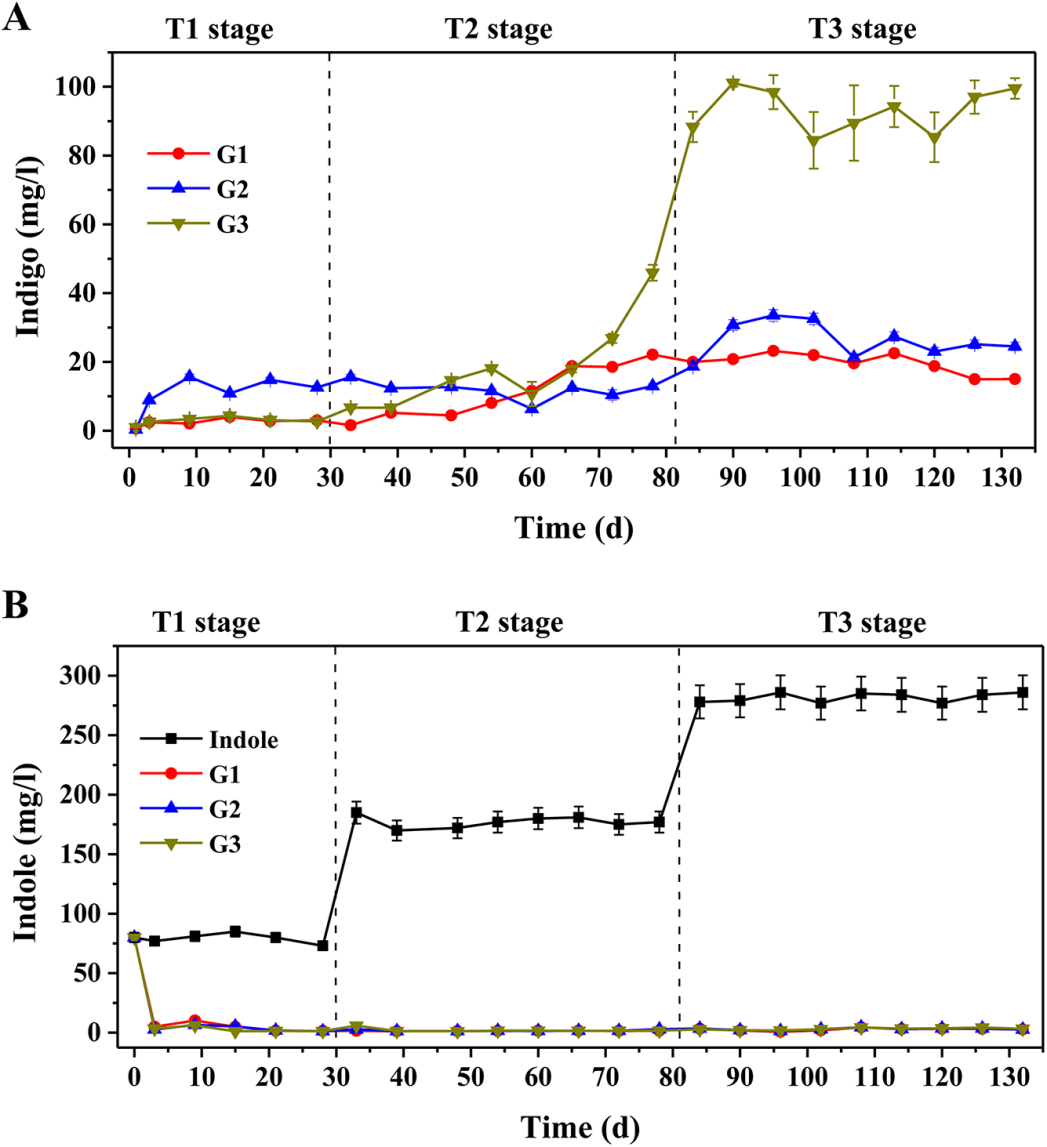
Indigo production from indole by microbial communities. **A.** Indigo production by three activated sludge systems. **B.** Indole consumption by three activated sludge systems. G1, non-augmented AS; G2, AS plus *Comamonas* sp. MQ; G3, AS plus *E*. *coli*_*nagAc*_. The concentrations of indole and indigo were measured by HPLC at the end of SBR operation cycle.

Three AS systems for indigo production were constructed using naphthalene and indole as the inducible substrates (Table A in S1 File). A blue product was produced by three reactors in the presence of indole. Metabolite analysis by HPLC-MS showed that the blue product had a prominent molecular ion (MH^+^) peak at m/z 263, which was confirmed to be indigo (Fig A in S1 File). The results demonstrated that all three AS systems were able to produce indigo from indole. Fig 1 depicts the performances of indigo production and indole consumption over the 132-day operation, which have been divided into three stages based on the changes in indole concentration, i.e. T1 (0–30 d), T2 (30–81 d) and T3 (81–132 d). In T1 stage, G2 produced the highest yields of indigo (nearly 15.7 ± 0.8 mg/l) compared to negligible production in G1 and G3. Subsequently, the indigo yields in G1 (17.3 ± 0.9 mg/l) and G3 (45.9 ± 2.3 mg/l) displayed modest increases in the T2 stage. All three treatments exhibited better capabilities for indigo production in T3 stage when the influent indole concentration was raised to 280 mg/l. After Day 90, indigo yields dramatically increased to approximately 99.5 ± 3.0 mg/l in G3, whereas the yields of G1 and G2 were only 19.2 ± 1.2 and 27.3 ± 1.3 mg/l, respectively. Indole was completely transformed at the end of each SBR cycle over the entire period of operation (Fig 1B). In the pure culture controls, strain MQ could produce 33.2 ± 2.5 mg/l indigo from indole at the first operation cycle due to the good activities of naphthalene dioxygenase induced in the cells (Fig B in S1 File). Similarly, the recombinant *E*. *coli*_*nagAc*_ could produce 68.4 ± 3.1 mg/l indigo from indole, while the non-recombinant *E*. *coli* without *nag* gene was unable to produce indigo (Fig B in S1 File). Then, strain MQ was able to grow in the synthetic wastewater medium when indole concentration was below 100 mg/l, but low yields of indigo (<10 mg/l) were produced due to the toxicity of indole to bacterial strains (Fig B in S1 File). When the concentrations of indole increased to above 100 mg/l, strain MQ could hardly grow. Whereas, the recombinant *E*. *coli*_*nagAc*_ was unable to grow due to the lack of nutrients and almost no indigo was produced after the first operation cycle. These results indicated that both inoculated strains might contribute to indigo production in the AS systems at early days of operation process. The differences in indigo production among the three treatments suggested the harbored microbial communities were the driving force in indigo production.

### Overview of sequencing analysis of 16S rRNA gene amplicons

A total of 1,721,342 effective reads with an average length of 253 bp were obtained from 36 samples, resulting in 1,422 OTUs at 97% sequence identity cutoff. Even at high depth (~27,500 sequences per sample) of sequencing, the rarefaction curves did not approach saturation, indicating that some microbial taxa remained undetected (Fig C in S1 File). Microbial richness (the observed OTU number and Chao1 estimator) was similar among the samples from G1, G2 and G3 (ANOVA, *P* > 0.05) (Table B in S1 File). However, both Shannon (H) and evenness (J) indices were significantly different among the three treatments in T1 stage (*P* < 0.05), probably owing to the inoculation of strain MQ and *E*. *coli*_*nagAc*_ at the beginning of operation. The H and J indices showed little difference in T2 stage (*P* > 0.05), but then became distinctly different in T3 stage (*P* < 0.05) (Table B in S1 File). Compared to the original sludges, the microbial richness and evenness were significantly lower in all three treatments (*P* < 0.001), leading to lower Shannon indices (Table B in S1 File), which indicated that the addition of indole reduced microbial diversity in the AS systems.

### Survival of the inoculated strains in the AS systems

The relative abundances of *Comamonas* sp. and *E*. *coli* were investigated to determine the survival rates of the inoculated strains. In G1 and G3, *Comamonas* sp. maintained about 40% of the population throughout operation process (Fig D in S1 File). However, in G2, before Day 10, 87% of the sequences belonged to *Comamonas* sp. (Fig D in S1 File), most likely due to the inoculation of *Comamonas* sp. MQ. From Day 10 to 30, the proportion of *Comamonas* sp. declined to around 30% and remained at that level (Fig D in S1 File). In contrast, very few *E*. *coli* sequences were detected in any of the groups based on taxonomic classification by the RDP classifier.

**Fig 2:**
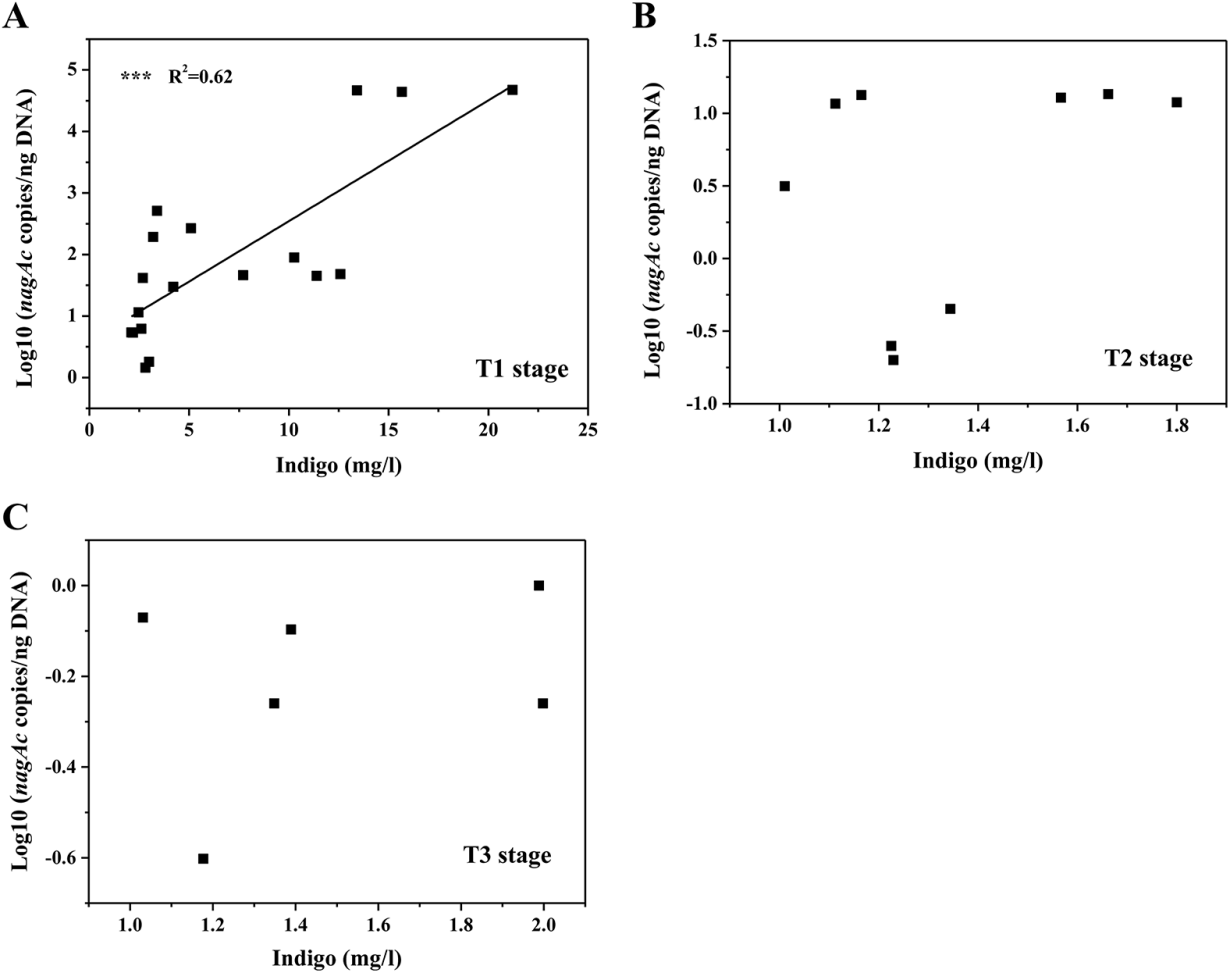
Relationship between the indigo yield and the abundance of *nagAc* gene in T1 (A), T2 (B) and T3 (C) stages. The line represents the linear-fit model to the data. Triple asterisks indicate *P* < 0.001. The relative abundance of the *nagAc* gene, given in log10 unit, was determined by qPCR quantification using the *nagAc* gene from *Comamonas* sp. MQ as the standard.

The *nagAc* gene from both inoculated strains was detected by qPCR at each sampling time point. In the non-augmented G1, there were less than 10 copies/ng *nagAc* at all the time points (Fig E in S1 File). In G2, the gene abundance peaked at Day 10, reaching 4.5×10^4^ copies/ng, which corresponded well with the high proportion of *Comamonas* sp. as shown above, yet dropped to 45 copies/ng at Day 30, and below 1 copy/ng at Day 130 (Fig E in S1 File). This gene was also detected in G3 with relatively high abundance within 10 days (200–500 copies/ng) (Fig E in S1 File). As indicated above, the relative abundances of *Comamonas* sp. in G1 and G3 were almost the same (Fig E in S1 File), but the *nagAc* abundances in G2 and G3 was much higher than those in G1. Thus, the *nagAc* gene in G3 should be from *E*. *coli*_*nagAc*_ added at the beginning, while the gene in G2 was probably from *Comamonas* sp. MQ. The indigo yields significantly correlated with the abundance of the *nagAc* gene (in log10 unit) in the T1 stage (R^2^ = 0.62, *P* < 0.001), but showing no significant relationship at the T2 and T3 stages (*P* > 0.10) (Fig 2). Based on the combination analyses of high-throughput sequencing and qPCR, it suggested that the inoculated strain MQ could remain in the AS systems for at least 30 days.

### Community composition and dynamics of three AS systems

**Fig 3:**
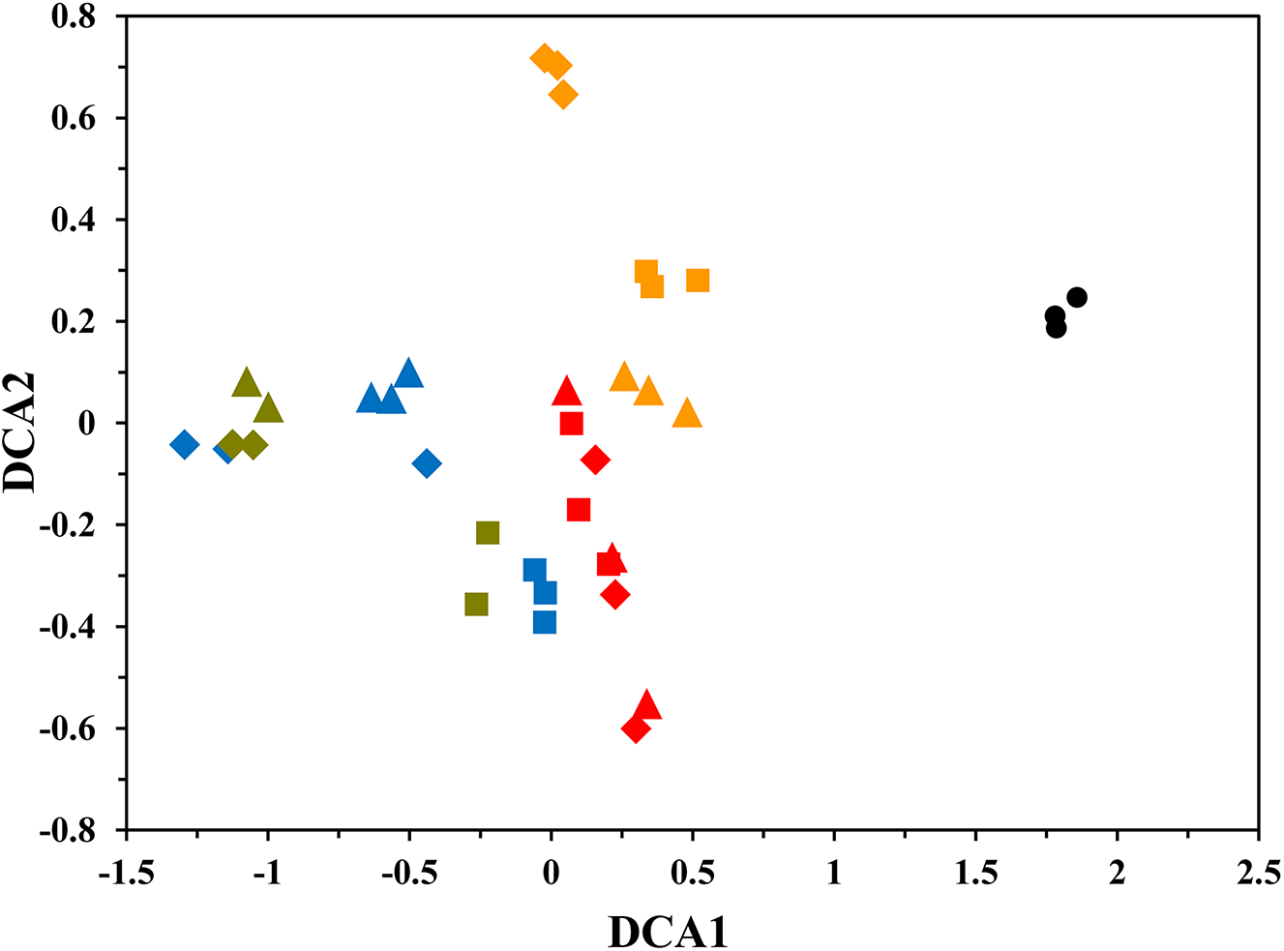
Detrended correspondence analysis (DCA) of microbial communities based on Illumina MiSeq sequencing. Symbols represented the samples from different treatments: cycle (●), original AS; square (■), G1; diamond (♦), G2; triangle (▲), G3. Colors represented the samples collected at different stages: orange, early days of T1 stage (0–15 d); red, late days of T1 stage (15–30 d); blue, T2 stage (30–81 d); green, T3 stage (81–132 d). Detailed group setup was presented in Table A in S1 File.

DCA was performed to visualize the succession of microbial communities over time. The microbial communities from three treatments diverged from the original AS after receiving indole (Fig 3). The three treatments clustered separately in early days of T1 stage (Day 6 to 12), but converged by late days of T1 stage (Day 24 to 30) (Fig 3), during which the inoculated strains had almost disappeared. Then in T2 stage, the groups separated, forming three clusters (Fig 3). In T3 stage, G2 and G3 were clustered, but distinctly separated from G1 (Fig 3). Results of three dissimilarity tests, Adonis, ANOSIM and MRPP, suggested that the community structures of the original AS and the three treatments were significantly different (*P* < 0.05) (Table C in S1 File). Furthermore, the communities of the three treatments were also distinctly different between T1 and T2 stages (*P* < 0.05), but not between T2 and T3 stages (*P* > 0.05) (Table C in S1 File).

**Fig 4:**
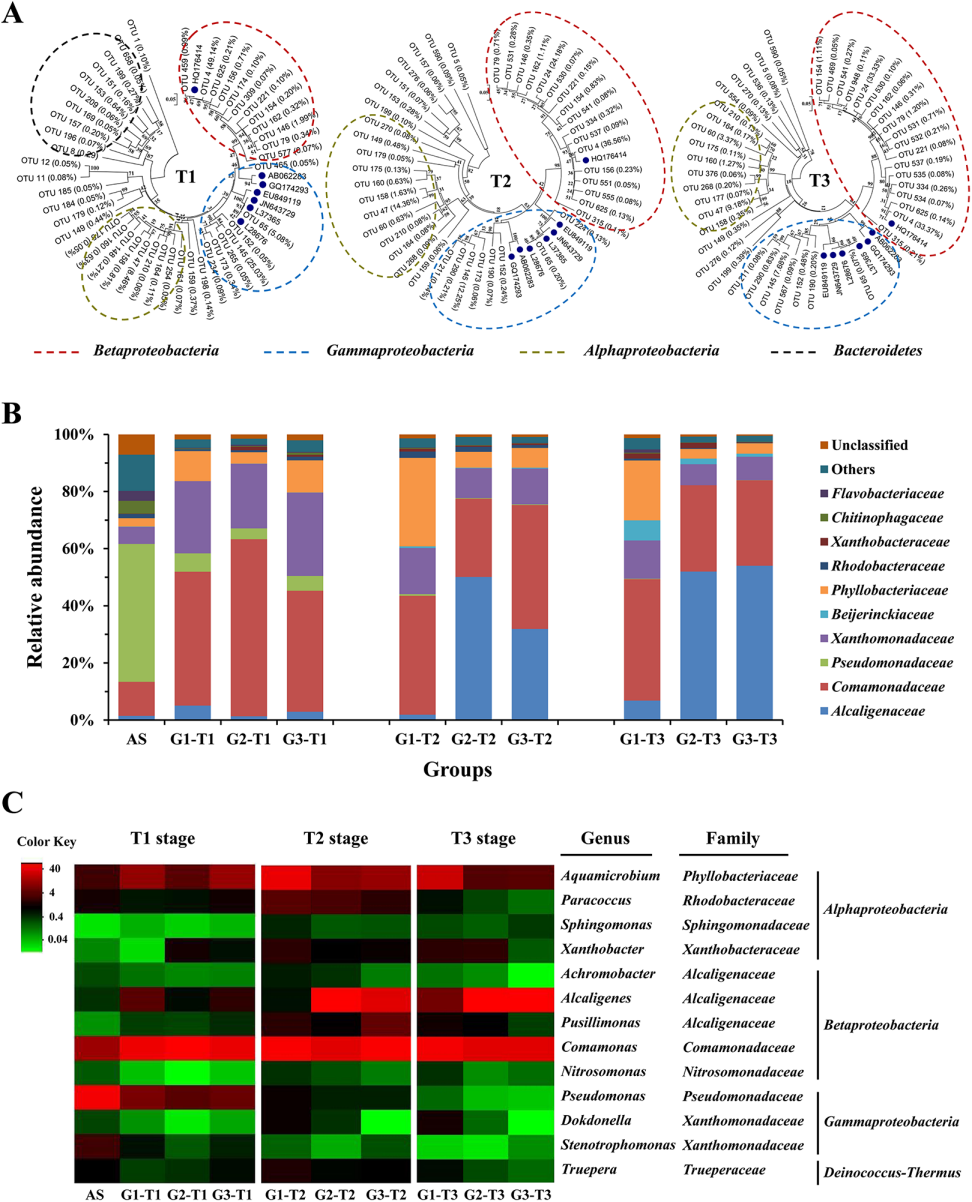
Community compositions of three treatments in T1, T2 and T3 stages. **A.** Phylogenetic tree of the shared 16S rRNA gene sequences constructed by the neighbor-joining method with 1,000 bootstrap replicates. The number in parentheses represented the relative abundances of each OTU, and only OTUs accounting for more than 0.05% of the shared sequences were shown. Sequences of typical indigo-producing strains reported previously were indicated with symbol (●) and the accession number. **B.** Relative abundance of the dominant families from the shared OTUs. **C.** Heat map of the 10 most abundant genera in each treatment. The color intensity in each cell showed the relative abundance of a genus in a treatment.

The microbial community composition of the three treatments are illustrated in Fig 4. Among the total 1,422 OTUs detected, 351, 197 and 143 OTUs were shared by the three treatments in T1, T2 and T3 stages, respectively (Fig F in S1 File). These shared OTUs accounted for 99% of the classified sequences in each stage. As a result, the downstream analysis focused primarily on the shared OTUs. Although 16 phyla were detected in T1 stage, *Proteobacteria* and *Bacteroidetes* covered over 97% of the shared sequences (Fig 4A). In T2 and T3 stages, more than 96% of the shared sequences belonged to *Proteobacteria* (Fig 4A), among which *Betaproteobacteria* was the most dominant class (43.8–77.5% in T2 stage, 49.7–84.0% in T3 stage). Compared with the three treatments, the original sludge communities had higher percentages of *Gammaproteobacteria* (54.8%, compared to 22.4% on average) and *Bacteroidetes* (14.0%, compared to 1.6% on average).

A total of 144 families were classified based on the RDP database and 87 families were shared by three treatments. Ten of these families represented over 90% of sequences. In the original AS, *Pseudomondaceae* was the most abundant and accounted for 48.3% of the sequences, followed by *Comamonadaceae* (11.9%), *Xanthomonadaceae* (6.0%), *Chitinophagaceae* (4.5%), *Flavobacteriaceae* (3.5%), *Phyllobacteriaceae* (2.9%), *Alcaligenaceae* (1.5%) and *Rhodobacteraceae* (1.4%) (Fig 4B). The community compositions of G1, G2 and G3 were distinct from the original AS (Fig 4B). For instance, *Comamonadaceae* was detected in all three treatments with considerably higher relative abundance (more than 40% on average). In G2, *Comamonadaceae* was extremely enriched in early days of T1 stage (83–90%) due to the addition of *Comamonas* sp. MQ, yet the abundance decreased in T2 and T3 stages and was even lower than those in G1 and G3 (Fig 4B). The abundance of *Pseudomonadaceae* decreased dramatically in T1 stage (3.7–6.5%) and further decreased in T2 (below 0.6%) and T3 (below 0.2%) stages. In contrast, the abundance of *Alcaligenaceae* was below 5% in T1 stage of each treatment, while increased to above 30% in T2 and T3 stages of G2 and G3, becoming the predominant population (Fig 4B). The relative abundance of *Phyllobacteriaceae* also increased in all three treatments (4.3–18.0%), especially G1 (10.5–31.0%). The most abundant genera from the original AS and three treatments are shown in Fig 4C. Four genera were abundant (> 1% on average) in all three treatments, i.e. *Comamonas* (family *Comamonadaceae*), *Pseudomonas* (family *Pseudomonadaceae*), *Alcaligenes* (family *Alcaligenaceae*) and *Aquamicrobium* (family *Phyllobacteriaceae*), which represented major fractions of their corresponding families (Table D in S1 File) and therefore shared similar dynamic patterns.

### Linking community composition to indigo production

**Fig 5:**
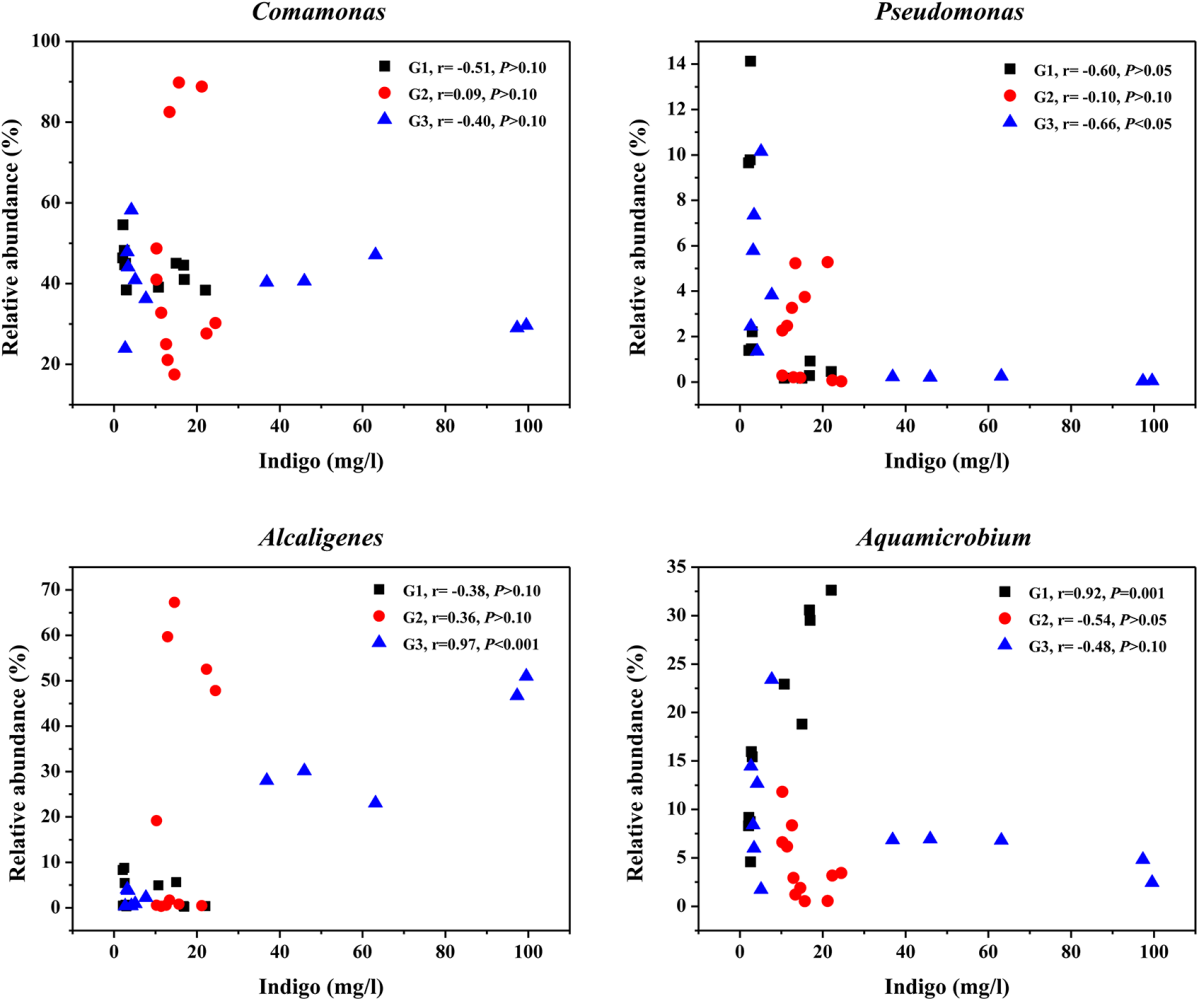
Correlations between the relative abundances of the major genera and indigo yield in each treatment. Pearson correlation coefficients (r) with the associated *P* values were shown for each taxon of each treatment.

To discern the relationship between community composition and indigo production, Pearson correlation test was conducted at both genus and family levels (Fig 5). Among the four major genera (*Comamonas*, *Pseudomonas*, *Alcaligenes and Aquamicrobium*), the most abundant genus *Comamonas* had no significant correlation with indigo yields in all groups (*P* > 0.10) (Fig 5). The abundance of *Pseudomonas* did not have correlations with indigo yields in G1 or G2 (*P* > 0.05) either, but was negatively correlated with indigo yields in G3 (r = -0.66, *P* < 0.05) (Fig 5). In contrast, the relative abundance of *Alcaligenes* exhibited a strong positive relationship with indigo yields in G3 (r = 0.97, *P* < 0.001), and the relative abundance of *Aquamicrobium* was also positively correlated with indigo yields in G1 (r = 0.92, *P* = 0.001) (Fig 5). The relative abundances of family *Comamonadaceae*, *Pseudomonadaceae*, *Alcaligenaceae* and *Phyllobacteriaceae* showed similar correlations with indigo yields as the corresponding genus in general (*P* < 0.05) (Fig G in S1 File).

The correlation analysis was also performed with other dominant families with more diverse genera. The relative abundances of *Rhodobacteraceae* and *Xanthobacteraceae* were positively correlated with indigo yields in G1 (*P* < 0.05), whereas the abundances of *Beijerinckiaceae* showed positive effects on indigo production in G2 and G3 (*P* < 0.05) (Fig G in S1 File). In contrast, the abundances of *Xanthomonadaceae* decreased with indigo yields in G1 and G3 (*P* < 0.05) (Fig G in S1 File).

## Discussion

While the microbial production of indigo has been comprehensively studied using wild strains or GEMs, less has been done to characterize the indigo-producing capacity of microbial communities. In this study, we successfully achieved indigo production from indole by AS and bioaugmented AS, and the associated microbial communities were revealed by Illumina MiSeq sequencing technology. The two bioaugmented groups produced higher yields of indigo (27.3 ± 1.3 and 99.5 ± 3.0 mg/l) compared with the non-augmented AS (19.2 ± 1.2 mg/l). Sequencing analysis revealed that the inoculated *Comamonas* sp. MQ was dominant early in the time course, while the recombinant *E*. *coli*_*nagAc*_ was not detected in the bioaugmented system. Similar results were observed in bioaugmentation studies on 3-chloroaniline and polyurethane biodegradation, in which the inoculated strains accelerated the removal of undesired compounds, but did not remain in activated sludge or soil communities [23,38,39]. qPCR analysis showed that the indigo yields were closely associated with *nagAc* gene abundance in T1 stage (Fig 2), and thus naphthalene dioxygenase might be responsible for the production of indigo. However, the abundance of *nagAc* gene was greatly reduced by Day 80 (Fig E in S1 File), and it had no significant relationship with indigo yields in T2 and T3 stages (Fig 2). The indigo yields, by contrast, increased significantly after Day 70, especially in G3 (Fig 1). Therefore, we would expect other oxygenases in the AS systems that might catalyze the biotransformation of indole to indigo, and the indigenous bacteria were primarily responsible for the high yields of indigo in the late period of operation. These results were similar to those reported by Bai et al. [25], who found that indigenous bacteria played the most significant roles in degrading pyridine and quinoline in their bioaugmented systems.

Based on DCA ordination and dissimilarity tests, the communities from the three treatment groups changed when indole was added. Both deterministic and stochastic processes could be involved in shaping the assembly and succession of the communities [40–43]. The separation of the three treatments in early days of T1 stage could be due to the addition of exogenous strains *Comamonas* sp. MQ and *E*. *coli*_*nagAc*_. But in late days of T1 stage, both inoculated strains were almost gone (Fig D in S1 File), and each treatment started to recover their own communities, during which the stochastic processes of colonization and extinction might be involved [41,43]. Thereafter, distinct communities had been established in T2 and T3 stages, and each community was assembled under the high indole pressure. A recent study by Zhou et al. [42] found that, even under identical conditions, the same source community could evolve into different communities with different structures and distinct functions due to stochastic processes. Chase [40] also found that stochastic processes were likely to play a strong role in the assembly of high-productivity communities. Therefore, we speculated that the differences in indigo production among the three treatments in T2 and T3 stages might be a consequence of stochastic processes in community assembly.

Previous studies have shown that *Comamonadaceae* (genus *Comamonas*) [13], *Moraxellaceae* (genus *Acinetobacter*) [11,12], *Nocardiaceae* (genus *Rhodococcus*) [9], *Pseudomonadaceae* (genus *Pseudomonas*) [1,5–8] and *Sphingomonadaceae* (genus *Sphingomonas*) [9] were able to produce indigo from indole. However, only a few sequences of *Moraxellaceae*, *Nocardiaceae* and *Sphingomonadaceae* were detected in all three treatments. The dominant families *Alcaligenaceae*, *Phyllobacteriaceae*, *Beijerinckiaceae*, *Rhodobacteraceae* and *Xanthobacteraceae* have not been reported to participate in indigo bio-production, and owing to the fact that the abundances of those families were positively correlated with indigo yields, there may be some new indigo-producing strains in these treatments.

Among the bacterial strains reported to produce indigo, *Pseudomonas* sp. is most commonly studied [1,5–8]. Although *Pseudomonas* has been widely used as a model microorganism in the study of naphthalene degradation, a previous study showed that *Pseudomonas* was not the dominant degrader in naphthalene-amended soil microcosms [44]. Likewise, in this study, *Pseudomonas* abundance decreased during the operation in all three treatments, thus displaying no positive relationship with indigo production. In our previous study, *Comamonas* sp. MQ exhibited the ability to produce indigo from indole [13]. As indigo yields were significantly positively correlated with the abundance of *nag* gene from *Comamonas* sp. MQ in T1 stage, *Comamonas* could play an important role in indigo production in the early stage. However, *Comamonas* displayed no significant correlations with indigo yields over the whole period of operation (*P* > 0.10). Therefore, there were likely other populations that could also produce indigo from indole. *Alcaligenes* and *Aquamicrobium*, with the ability to degrade aromatic compounds [45,46], might serve as good candidates for indigo production in this study. During the past several decades, only one study found trace amounts of indigo in the culture broth of *Alcaligenes* sp. In3 when growing with indole [47]. While *Aquamicrobium* sp. was reported to be capable of degrading thiophene-2-carboxylate and biphenyls [46,48], indigo production has been rarely described. In the present study, the relative abundance of *Alcaligenes* increased dramatically in T2 and T3 stages of G3, during which the system reached the maximal indigo production. A significantly positive correlation was obtained between the abundance of *Alcaligenes* and indigo yields in G3 (Fig 5). Similarly, a strong positive correlation of relative abundance with indigo yields was also found for *Aquamicrobium* in G1 (Fig 5). In previous report [19], we have found that *Aquamicrobium* could serve as a new biocatalyst for indigo production in AS systems. Therefore, it was presumed that *Alcaligenes* and *Aquamicrobium* were likely to be the functional bacteria in the AS communities responsible for indigo production. The present study also suggests that there are more bacteria capable of producing indigo from indole as revealed by high-throughput sequencing technologies. Nevertheless, the duplicate measurements in a single reactor run does not make up for the lack of a second or third replicate reactor with the exact same conditions, and further investigation needs to be carried out in triplicate in order to provide a more effective analysis of microbial communities.

In summary, the possibility of bioaugmented microbial communities producing indigo was demonstrated, and our studies also supplied the Illumina sequencing data for the microbial communities associated with indigo production during 132-day operation. The structure and diversity of the communities changed greatly over time. Our results suggested several populations might participate in indigo production, such as *Alcaligenes* and *Aquamicrobium*, which had not been widely observed previously. This study should provide important information on microbial communities for the production of indigo from indole.

## Supporting Information

S1 File This includes Tables A-D and Figures A-G. Table A. Experimental setup and sampling in this study. Table B. Diversity indices of the original AS and three treatments. Table C. Dissimilarity tests of microbial communities from three treatments at different stages. Table D. Relative abundances of the major genera (>1% on average) in the original AS and three treatments. Fig A. Mass spectra of the products produced by activated sludge systems. Fig B. Indigo production performance of the pure culture controls. Fig C. Rarefaction curves based on 16S rRNA gene amplicon sequencing of microbial communities. Fig D. Relative abundance of *Comamonas* sp. in the original AS and three treatments. Fig E. Relative abundance of *nagAc* gene in the original AS and three treatments. Fig F. Venn diagrams based on all detected OTUs at a cutoff of 3% sequence similarity. Fig G. Correlations between the relative abundances of major phylotypes and indigo yields in each treatment.
(PDF)
