# Supplementary material for: Microbial Community Dynamics and Activity Link to Indigo Production from Indole in Bioaugmented Activated Sludge Systems
Source: PLoS One. 2015 Sep 15;10(9):e0138455. doi: 10.1371/journal.pone.0138455 (PMC4570806; doi:10.1371/journal.pone.0138455)
Supplement: S1 File — Table A. Experimental setup and sampling in this study. Table B. Diversity indices of the original AS and three treatments. Table C. Dissimilarity tests of microbial communities from three treatments at different stages. Table D. Relative abundances of the major genera (>1% on average) in the original AS and three treatments. Fig A. Mass spectra of the products produced by activated sludge systems. Fig B. Indigo production performance of the pure culture controls. Fig C. Rarefaction curves based on 16S rRNA gene amplicon sequencing of microbial communities. Fig D. Relative abundance of Comamonas sp. in the original AS and three treatments. Fig E. Relative abundance of nagAc gene in the original AS and three treatments. Fig F. Venn diagrams based on all detected OTUs at a cutoff of 3% sequence similarity. Fig G. Correlations between the relative abundances of major phylotypes and indigo yields in each treatment. (PDF) [file pone.0138455.s001.pdf]

Supporting Information for *PLoS ONE*

Microbial community dynamics and activity link to indigo production from indole in bioaugmented activated sludge systems

Yuanyuan Qu[1,2*¶], Xuwang Zhang[1¶], Qiao Ma[1,2¶], Jie Deng[2], Ye Deng[2], Joy D. Van Nostrand[2], Liyou Wu[2], Zhili He[2], Yujia Qin[2], Jiti Zhou[1], Jizhong Zhou[2,3,4]

[1] State Key Laboratory of Fine Chemicals, Key Laboratory of Industrial Ecology and Environmental Engineering (Ministry of Education), School of Environmental Science and Technology, Dalian University of Technology, Dalian 116024, China

[2] Institute for Environmental Genomics (IEG), Department of Microbiology and Plant Biology, University of Oklahoma, Norman, Oklahoma 73019, USA

[3] Earth Sciences Division, Lawrence Berkeley National Laboratory, Berkeley, CA 94720, USA

[4] State Key Joint Laboratory of Environment Simulation and Pollution Control, School of Environment, Tsinghua University, Beijing 100084, China

*Corresponding author:

E-mail: qyy@dlut.edu.cn

¶These authors contributed equally to this work.

Table A. Experimental setup and sampling in this study.

Group	Time[a]	Samples	Conditions
AS	T0	M1, M2, M3	Original activated sludge
G1	T1[b]	M4, M8, M12, M16, M20, M24	Using AS directly
	T2	M28, M32, M36	
	T3	M40, M44	
G2	T1	M5, M9, M13, M17, M21, M25	Using AS plus strain MQ
	T2	M29, M33, M37	
	T3	M41, M45	
G3	T1	M6, M10, M14, M18, M22, M26	Using AS plus *E. coli* $_{nagAc}$
	T2	M30, M34, M38	
	T3	M42, M46	

[a]T0 stands for 0th day of operation process before indole was added; T1 for 0-30 days, and samples are collected at 6th, 9th, 12th, 24th, 28th and 30th day; T2 for 30-81 days, and sample are collected at 75th, 78th and 81st day; T3 for 81-132 day, and samples are collected at 129th and 132th day.

[b]T1 can be divided into early and late days of operation: early days include 6th, 9th and 12th day; late days include 24th, 28th and 30th day.

Table B. Diversity indices of the original AS and three treatments.

Groups	Time	Shannon index (H)	Evenness (J)	Chao1	OTUs
AS	T0	2.68 ±0.12	0.44 ±0.02	555 ±21	444 ±11
	T1	1.82 ±0.23	0.32 ±0.04	407 ±69	284 ±35
G1	T2	1.81 ±0.04	0.32 ±0.01	438 ±23	276 ±12
	T3	2.13 ±0.02	0.38 ±0.01	339 ±19	262 ±7
	T1	1.31 ±0.54	0.24 ±0.08	338 ±115	239 ±73
G2	T2	1.68 ±0.34	0.30 ±0.04	361 ±81	275 ±91
	T3	1.72 ±0.04	0.32 ±0.01	359 ±17	224 ±12
	T1	1.89 ±0.20	0.33 ±0.03	419 ±51	287 ±35
G3	T2	1.90 ±0.02	0.34 ±0.01	399 ±39	260 ±11
	T3	1.75 ±0.05	0.33 ±0.01	338 ±43	209 ±5

Table C. Dissimilarity tests of microbial communities from three treatments at different stages.[a]

Data sets	Adonis[b]		ANOSIM[c]		MRPP[d]	
	R^2	*P*	R	*P*	δ	*P*
AS vs. Three treatments	0.297	0.001	0.964	0.001	0.38	<0.001
T1 stage vs. T2 stage	0.285	0.001	0.477	0.001	0.317	<0.001
T2 stage vs. T3 stage	0.113	0.197	0.091	0.168	0.327	0.110

[a]Different statistical approaches were used with Bray-Curtis distances, and *P* values were of corresponding significance tests.

[b]Adonis, permutational multivariate analysis of variance with the Adonis function.

[c]ANOSIM, analysis of similarity.

[d]MRPP, multiresponse permutation procedure.

Table D. Relative abundances of the major genera (>1% on average) in the original AS and three treatments.[a]

Time	Proteobacterial class	Family	Genus	AS	G1	G2	G3
T1	*Betaproteobacteria*	*Comamonadaceae*	*Comamonas*	11.75 (99%)	46.39 (99%)	61.67 (99%)	42.05 (99%)
	Gammaproteobacteria	*Pseudomonadaceae*	*Pseudomonas*	48.29 (100%)	6.46 (100%)	3.73 (100%)	5.17 (100%)
	Betaproteobacteria	*Alcaligenaceae*	*Alcaligenes*	0.38 (26%)	3.99 (79%)	0.73 (55%)	1.98 (68%)
	Alphaproteobacteria	*Phyllobacteriaceae*	*Aquamicrobium*	2.48 (85%)	10.39 (99%)	3.92 (98%)	11.14 (99%)
T2	*Betaproteobacteria*	*Comamonadaceae*	*Comamonas*	-	41.29 (99%)	26.44 (97%)	42.64 (98%)
	Gammaproteobacteria	*Pseudomonadaceae*	*Pseudomonas*	-	0.54 (100%)	0.22 (100%)	0.22 (100%)
	Betaproteobacteria	*Alcaligenaceae*	*Alcaligenes*	-	0.31 (16%)	48.80 (97%)	27.04 (85%)
	Alphaproteobacteria	*Phyllobacteriaceae*	*Aquamicrobium*	-	30.83 (100%)	5.49 (99%)	6.84 (99%)
T3	*Betaproteobacteria*	*Comamonadaceae*	*Comamonas*	-	42.07 (99%)	28.89 (96%)	29.30 (98%)
	Gammaproteobacteria	*Pseudomonadaceae*	*Pseudomonas*	-	0.16 (100%)	0.04 (100%)	0.04 (100%)
	Betaproteobacteria	*Alcaligenaceae*	*Alcaligenes*	-	5.26 (77%)	50.16 (96%)	48.82 (90%)
	Alphaproteobacteria	*Phyllobacteriaceae*	*Aquamicrobium*	-	20.80 (99%)	3.27 (97%)	3.62 (99%)

[a]The percentage of each genus in its corresponding family was shown in the parenthesis.

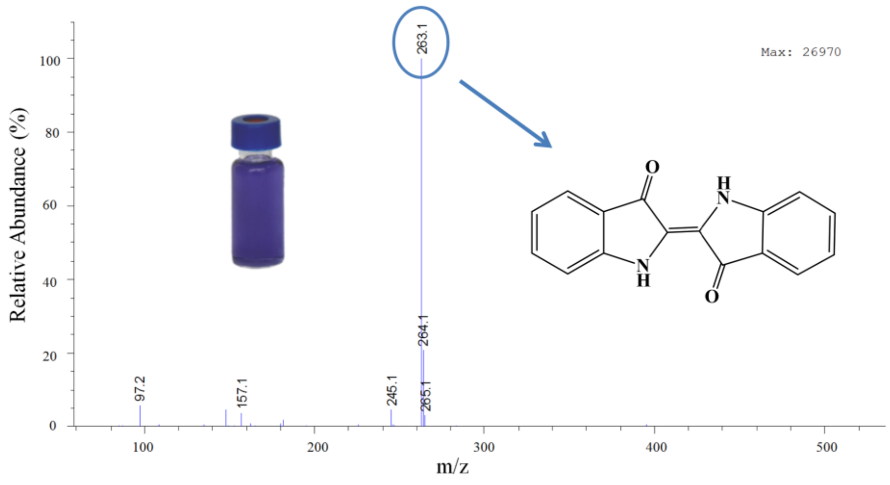

Fig A. Mass spectra of the products produced by activated sludge systems.

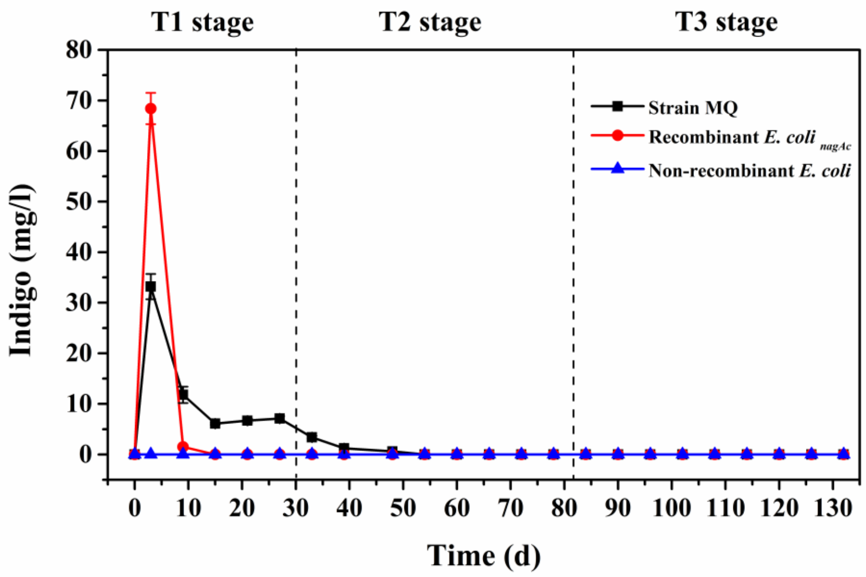

Fig B. Indigo production performance of the pure culture controls. The assays were performed by inoculating the same amount of strain MQ and recombinant *E. coli* $_{nagAc}$ into the synthetic wastewater, respectively, and the operation processes were carried out under the identical conditions as the AS systems. T1 stage (0-30 d), indole was 73-85 mg/l; T2 stage (30-81 d), indole was 168-185 mg/l; and T3 stage (81-132 d), indole was 277-290 mg/l.

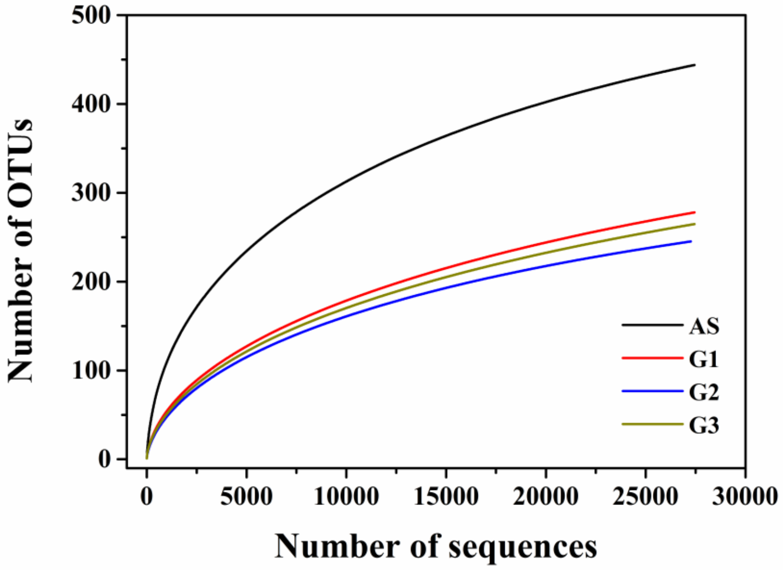

Fig C. Rarefaction curves based on 16S rRNA gene amplicon sequencing of microbial communities. The OTUs were defined by 3% distances.

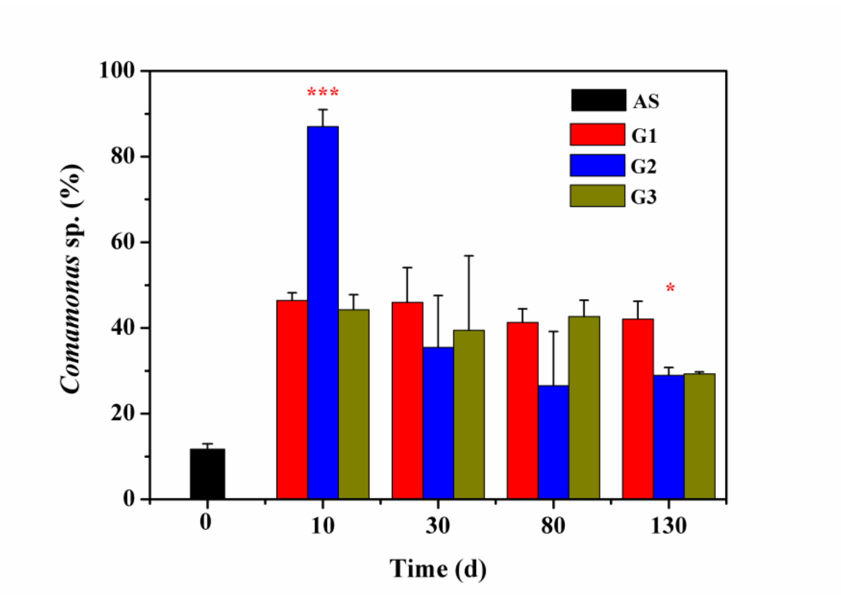

Fig D. Relative abundance of *Comamonas* sp. in the original AS and three treatments. The data were plotted using the average values obtained at each periods from the original activated sludge (AS; black), group 1 (G1; red), group 2 (G2; blue) and group 3 (G3; green). *: $P < 0.05$; ***: $P < 0.001$.

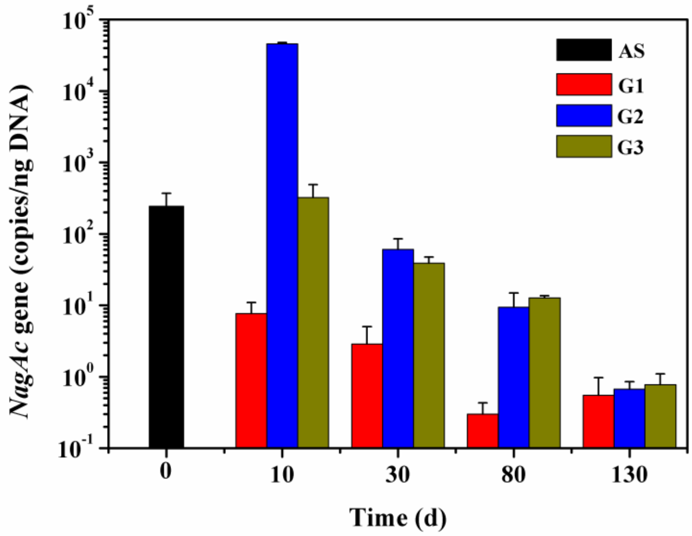

Fig E. Relative abundance of *nagAc* gene in the original AS and three treatments. The data were determined by RT-PCR quantification using the *nagAc* gene from *Comamonas* sp. MQ as standard, and plotted using the average values obtained at each periods from the original activated sludge (AS; black), group 1 (G1; red), group 2 (G2; blue) and group 3 (G3; green).

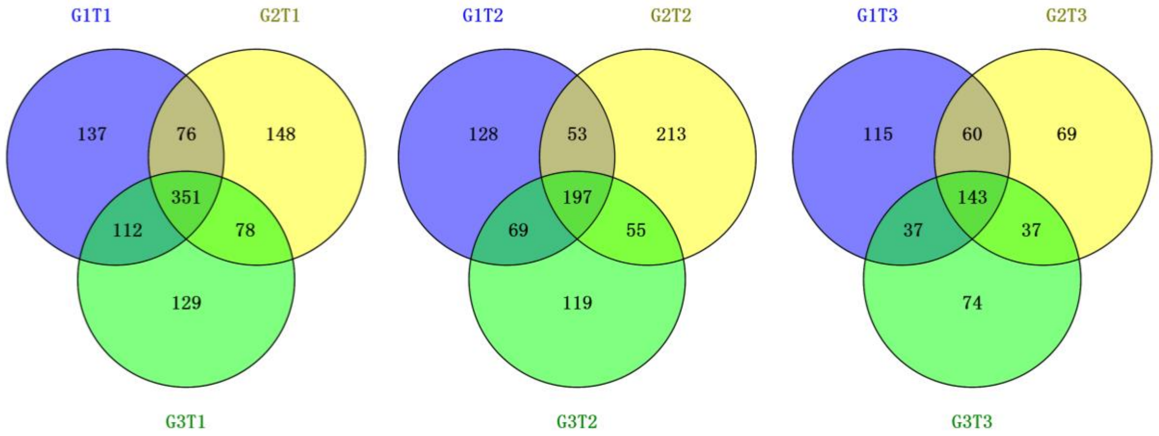

Fig F. Venn diagrams based on all detected OTUs at a cutoff of 3% sequence similarity. The shared OTUs accounted for 99% of the classified sequences in each stage. The Venn diagrams were generated by Venny 2.0 (http://bioinfogp.cnb.csic.es/tools/venny/index.html).

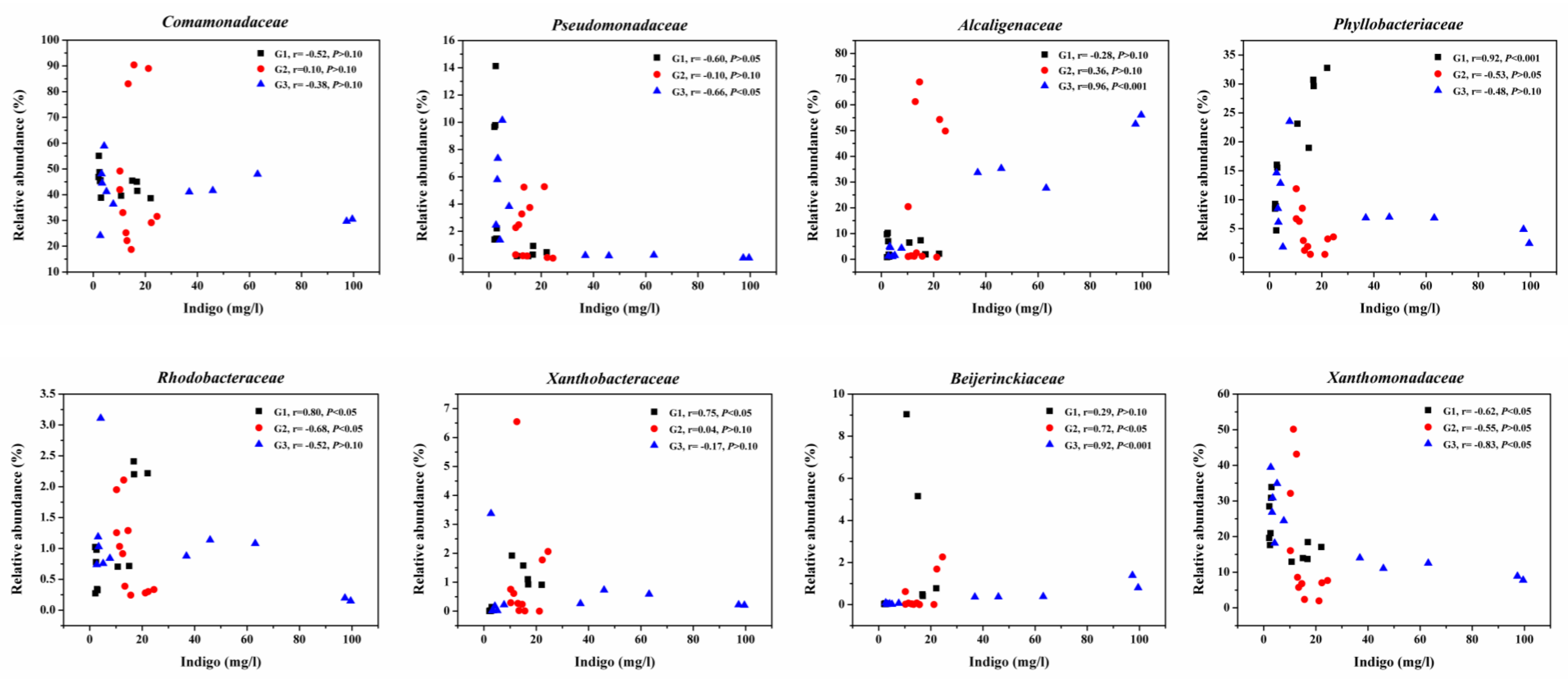

Fig G. Correlations between the relative abundances of major phylotypes and indigo yields in each treatment. Pearson correlation coefficients (r) with the associated *P* values were shown for each taxon of each treatment.
